# The effect of natalizumab on disability score and relapse rate of multiple sclerosis patients: a prospective cohort study

**DOI:** 10.1186/s40169-018-0216-3

**Published:** 2018-11-30

**Authors:** Mehrdokht Mazdeh, Shno Hosseini, Mohammad Taheri, Soudeh Ghafouri-Fard

**Affiliations:** 1 Department of Neurology, Hamadan University of Medical Sciences, Hamadan, Iran; 2 Neurophysiology Research Center, Hamadan University of Medical Sciences, Hamadan, Iran; 3 Student Research Committee, Shahid Beheshti University of Medical Sciences, Tehran, Iran; 4 Department of Medical Genetics, Shahid Beheshti University of Medical Sciences, Tehran, Iran

**Keywords:** Natalizumab, Multiple sclerosis, Relapse, EDSS

## Abstract

Multiple sclerosis (MS) is a progressive immune-related disorder of the central nervous system leading to destruction of myelin sheaths. Natalizumab is a humanized monoclonal antibody against the cell adhesion molecule α4-integrin which has been approved for treatment of relapsing forms of MS. This study aims at determining the effect of natalizumab on expanded disability status scale (EDSS) score and relapse rate of MS patients. Fifty MS patients participated in the present prospective cohort study. Twenty patients (Mean age ± SD: 33 ± 6.03) received natalizumab and 30 patients (Mean age ± SD: 36.83 ± 7.24) were under treatment with IFN-β (control group). Patients were followed-up during a 12-month period. EDSS score and clinical signs were assessed monthly. Significant decreases were detected in EDSS score in natalizumab treated patients compared with the controls in months 10, 11 and 12. EDSS score showed a significant decrease in 80% of natalizumab treated patients. Number of relapses was significantly lower in natalizumab treated patients compared with control group. Natalizumab is effective in improvement of disability and reduction of relapse rate in MS patients.

## Introduction

Multiple sclerosis (MS) is a chronic inflammatory disorder of the central nervous system (CNS) caused by both inflammatory harm and neurodegeneration [1]. Lymphocyte activation [2] and clonal expansion of T cell subpopulations [3] have crucial roles in the pathology of this disorder. As lymphocytes relocation through the blood–brain barrier (BBB) is an important step in their gathering in the CNS, a promising therapeutic strategy of MS relies on inhibition of such lymphocyte migration [1]. Relocation of lymphocytes across BBB is mediated through their binding with cell adhesion molecules such as vascular cell adhesion molecule-1 (VCAM-1) which are expressed on the endothelial cells [4]. VCAM-1 binds to α4β1 integrin which is expressed on almost all leukocytes subpopulations. Obstruction or prevention of VCAM-1/α4β1 interaction has been suggested as a therapeutic option for a wide spectrum of autoimmune disorders for a long time [5]. Considering the role of α4 integrin in enhancement of lymphocytes binding to the endothelium and their subsequent activation and increased proliferation as well as triggering cytokine cascade, α4 integrin has been proposed as a therapeutic target in MS [1]. The reported success of antibodies against the α4β1 integrin in inhibition of lymphocytes gathering in the CNS and amelioration of the experimental autoimmune encephalomyelitis [6] has paved the way for evaluation of this strategy in human subjects. In this way, natalizumab (Tysabri^®^; Biogen Idec/Elan, Cambridge, MA, USA) as the leading α4 integrin antagonist received US Food and Drug Administration (FDA) approval for treatment of MS patients [1]. Afterwards, several groups have evaluated the effect of this drug as a solitary drug or in combination with other disease modifying therapies (DMTs) in MS patients [7, 8]. In the present prospective cohort study, we compared the effects of natalizumab and IFN-β in Iranian patients with relapsing–remitting MS (RRMS) during a 12-month period.

## Methods

The present study was a 12-month cohort study to evaluate the efficacy of monthly intravenous (IV) infusion of natalizumab (at a dose of 300 mg) in reduction of relapse rate and expanded disability status scale (EDSS) score of RRMS patients who were referred to Farshchian Hospital, Hamadan, Iran during 2015–2016. The study protocol was approved by the ethical committee of Hamadan University of Medical Sciences. All patients agreed to participate in the study after complete explanations about the study protocol. Written informed consent forms were obtained from all study participants. Samples were selected based on the availability sampling method. Patients were randomly allocated by a medical staff to receive natalizumab (study group, n = 25) or IFN-β (control group, n = 30). Sample size was estimated to be 40 (20 individuals in each group) based on the parameters obtained from Oliveria et al. study [9]:

D = µ1–µ2 = 0.1, α = 0.05, β = 20%, power = 80%, µ1 (relapse rate in study group) = 2.4, µ2 (relapse rate in control group) = 2.5.

Five patients in the study group left the study because of their personal reasons. Demographic data, disease duration, EDSS score and former therapeutic regimens were recorded from all study members. All study participants were diagnosed to have RRMS based on the revised McDonald criteria [10]. The inclusion criteria were age between 18 and 50, two confirmed relapses during the prior year despite IFN-β treatment, appropriate response to high-dose corticosteroid pulse therapy and EDSS score less than 6. Complete blood count (CBC), liver function test, thyroid function test and urine analysis (UA) were tested in all patients. Patients with primary or secondary progressive MS, history of other chronic disorders, cancer, pregnancy, previous administration of cyclophosphamide/mitoxantrone/monoclonal antibodies, chronic liver disease, elevation of liver enzymes/bilirubin/Alkaline phosphatase/Creatinine, white blood cell < 3500 or lymphocyte count < 800 were excluded from the study. Moreover, lack of response to corticosteroid pulse therapy was regarded as an exclusion criterion. Patients in control group received IFN-β [intramuscular injection of 20 μg of CinnoVex (CinnaGen Co, Tehran, Iran) three-times a week] during the study period. Patients visited the clinic every 4 weeks for assessment of the EDSS and any treatment-associated complication. CBC and UA were assessed for patients under treatment with natalizumab in every visit.

### Statistical analysis

SPSS version 16 was used for data analysis. Distribution of data was first assessed by the Kolmogorov–Smirnov test. Mean values of relapse rate and EDSS score were compared before and after treatment using paired T test and Wilcoxon test according to the normality of data. Independent T and Mann–Whitney tests were used for comparison of these values between two study groups. Association between categorical variables was evaluated using Chi square test. The relationships between quantitative variables were evaluated using Pearson or ANOVA tests. P values less than 0.05 were considered as significant. EDSS scores in each month were compared using ANOVA test.

## Results

There was no significant difference in age and sex ratio between two study groups [study group (Mean age ± SD): 33 ± 6.03, control group (Mean age ± SD): 36.83 ± 7.24, P = 0.056; female/male ratio of 24/6 and 16/4 respectively, X^2^ = 0.00, df = 1, P = 0.65]. The clinical signs and symptoms in each study group are summarized in Table 1.

**Table 1:** The clinical manifestations of disease in each study group

Clinical symptoms and signs	Study group Number (%)	Control group Number (%)
Blurred vision	3 (15)	8 (26.6)
Diplopia	4 (20)	4 (13.3)
Blindness	1 (5)	0 (0)
Gait problem	5 (25)	2 (6.6)
Paresthesia	3 (15)	2 (6.6)
Muscle weakness	0 (0)	3 (10)
Dysarthria	0 (0)	1 (3.3)
Bladder dysfunction	0 (0)	1 (3.3)

Disease duration was 9.1 ± 4.79 and 8.77 ± 2.5 in study and control groups respectively (t = 0.32, df = 48, P = 0.75). EDSS scores were measured in all study participants each month (Table 2). Based on the results of Kolmogorov–Smirnov test showing normal distribution of data, ANOVA test was used for comparison of mean values between two groups. EDSS scores were significantly higher in study group compared with control group at month 1–4. However, EDSS scores were meaningfully lower in study group compared with control group at months 10–12.

**Table 2:** EDSS scores in each month during the study period

EDSS scores	Groups	Mean	Standard deviation	Minimum	Maximum	F	Sig
Month 1	Study	5.45	0.15	5	5.5	14.46	0.00
	Control	4.52	1.09	3	6		
Month 2	Study	5.45	0.15	5	5.5	14.46	0.00
	Control	4.52	1.09	3	6		
Month 3	Study	5.45	0.39	5.4	6	11.93	0.00
	Control	4.55	1.12	3	6.5		
Month 4	Study	5.25	0.55	4	6	5.38	0.03
	Control	4.63	1.10	3	6.5		
Month 5	Study	5.10	0.68	4	6	0.99	0.33
	Control	4.83	1.06	3	6.5		
Month 6	Study	4.95	0.74	3	6	0.13	0.72
	Control	4.85	1.08	3	6.5		
Month 7	Study	4.85	0.75	3	5.5	1.00	0.96
	Control	4.87	1.15	3	7		
Month 8	Study	4.63	0.94	3	6	1.30	0.26
	Control	4.97	1.10	3	7		
Month 9	Study	455	0.93	3	6	3.74	0.06
	Control	5.13	1.11	3.5	7		
Month 10	Study	4.33	1.12	2.5	6	6.35	0.02
	Control	5.15	1.15	3	7		
Month 11	Study	4.13	1.35	2	6	7.63	0.01
	Control	5.12	1.17	3	7		
Month 12	Study	3.98	1.37	2	6	9.54	0.00
	Control	5.10	1.18	3	7		

Subsequently, we compared EDSS scores in both groups in each time intervals using paired t-test (Tables 3 and 4 respectively). EDSS score in month 12 was significantly different from EDSS scores of month 1 to month 7 both in natalizumab treated patients and in control group.

**Table 3:** EDSS scores in the study group in each time intervals

Time intervals	Paired difference			t	df	Sig (2-tailed)
	Mean	Standard deviation	95% confidence interval			
1–12	1.48	1.36	0.84, 2.11	4.84	19	< 0.001
2–12	1.48	1.34	0.85, 2.10	4.91	19	< 0.001
3–12	1.48	1.15	0.94, 2.01	5.72	19	< 0.001
4–12	1.28	1.04	0.79, 2.76	5.46	19	0.001
5–12	1.13	1.02	0.65, 2.60	4.91	19	0.016
6–12	0.98	0.98	0.52, 1.43	4.45	19	0.016
7–12	0.88	0.90	0.45, 1.30	4.34	19	0.006
8–12	0.65	0.71	0.32, 0.98	4.10	19	0.088
9–12	0.58	0.71	0.24, 0.91	3.61	19	0.601
10–12	0.35	0.59	0.08, 0.62	3.67	19	0.083
11–12	0.15	0.33	0.00, 0.30	2.04	19	0.032

**Table 4:** EDSS scores in the control group in each time intervals

Time intervals	Paired difference			T	df	Significance
	Mean	Standard deviation	95% confidence interval			
1–12	− 0.58	0.70	− 0.84, − 0.32	− 4.59	29	0.001
2–12	− 0.58	0.70	− 0.84, − 0.32	− 4.59	29	0.001
3–12	− 0.55	0.71	− 0.82, − 0.28	− 4.24	29	0.001
4–12	− 0.47	0.66	− 0.71, − 0.22	− 0.39	29	0.001
5–12	− 0.27	0.57	− 0.48, − .0.05	− 2.57	29	0.016
6–12	− 0.25	0.54	− 0.45, − 0.05	− 2.55	29	0.016
7–12	− 0.23	0.43	− 0.39, − 0.07	− 0.97	29	0.006
8–12	− 0.13	0.41	− 0.29, 0.02	− 1.77	29	0.088
9–12	− 0.03	0.35	− 0.10, 0.16	0.53	29	0.601
10–12	0.05	0.5	− 0.01, 0.11	0.80	29	0.083
11–12	0.02	0.09	− 0.02, 0.05	1.00	29	0.326

We also evaluated relapse rate in both groups throughout the 12-month study period (Table 5). Significant difference was noticed in the quantity of relapses between two groups (χ^2^ = 42.94, df = 1, P < 0.001). Time points of relapses were sixth and seventh months in the treatment group.

**Table 5:** Number of relapses in each study group during the 12-month study period

Number of relapses	Groups	
	Study group Number (%)	Control group Number (%)
0	18 (90)	0 (0)
1	2 (10)	11 (36.6)
2	0 (0)	19 (63.3)

Pearson correlation test showed no significant correlation between EDSS in month 12 and patients’ age either in natalizumab treated patients (r = 0.12, P = 0.60) or control group (r = 0.28, P = 0.13). No significant correlation was found between age and number of relapses in either groups (r = 0.27, P = 0.056). No significant difference was found in mean EDSS scores in month 12 between males and females in either groups (P = 0.64 and 0.07 in natalizumab treated patients and controls respectively). EDSS in month 12 was correlated with disease duration in control group (r = 0.55, P = 0.002) but not in natalizumab treated patients (r = − 0.15, P = 0.52). Number of relapses was not associated with disease duration in in natalizumab treated patients (P = 0.39). However, in control group patient with two relapses had higher disease duration (P = 0.04). Assessment of lesion load in MRI verified the efficacy of natalizumab as all patients experienced lowering of MS plaques after completion of treatment with natalizumab.

## Discussion

In the present study, we compared efficacy of natalizumab and IFN-β in reduction of relapse rate and EDSS score in a 12-month study period and found better results for natalizumab in both parameters. Ninety percent of patients treated with natalizumab experienced no relapses during the study period. Moreover, despite higher primary EDSS scores in natalizumab treated patients, EDSS score at month 12 was significantly lower in these patients compared with control group which further shows the efficacy of natalizumab. Polman et al. have previously shown the efficacy of natalizumab in decreasing the risk of the continuous progression of disability and the frequency of relapses in patients with RRMS [8]. Rudick et al. have administered natalizumab along with IFN-β in RRMS patients and reported superiority of this kind of treatment compared with IFN-β alone in patients with RRMS [7]. In a randomized, double-blind trial, Miller et al. have compared effectiveness of two doses of natalizumab (3 and 6 mg/kg) with placebo and found significant decreases in the mean number of new lesions in both natalizumab groups [11]. Assessment of patients referred to two Brazilian MS centers showed significant decrease in relapse rate and an improvement of neurological incapacity after 1 year treatment with natalizumab. The effect of this drug was better in patients with lower EDSS scores [9]. Therefore, our results in Iranian MS patients are consistent with the previous studies in other populations. Moreover, in the present study, all natalizumab-treated patients were unresponsive to former treatment with IFN-β as they experienced at least two relapses prior year. So IFN-β was discontinued in this group of patients. Consequently, our study provides further evidences for effectiveness of natalizumab as a single second-line treatment for RRMS patients.

In brief, we demonstrated efficacy of natalizumab in decreasing relapse rate and EDSS score regardless of patients’ age, sex or disease duration which potentiate this drug as a putative treatment for many MS patients.
